# Lipid inclusions accumulation in macrophages from peritoneal effusion of a newborn

**DOI:** 10.1002/jha2.124

**Published:** 2020-10-27

**Authors:** Elise Kaspi, Diane Frankel, Patrice Roll

**Affiliations:** ^1^ Aix Marseille Univ, APHM, INSERM, MMG, Hôpital la Timone, Service de Biologie Cellulaire Marseille 13005 France

A newborn boy presented with profuse peritoneal effusion. Fetal ascites had been diagnosed by antenatal ultrasound since the ninth week of amenorrhea and worsened until birth (38th week of amenorrhea). Fetal macrosomia was observed with no morphological abnormality and no cardiac etiology that could explain ascites. Fetal karyotype did not showed any anomalies. At birth, peripheral blood tests only exhibited lymphopenia (1.13 × 10^6^ cells*/*mL).

Peritonal effusion was punctured on the second day of life. Ascites cellular count was 8700 nuclear cells/μL, 900 red cells/μL, and cytological analysis (May‐Grünwald‐Giemsa stained) revealed a predominance of lymphocytes (91%), associated with a small contingent of macrophages (6%), some of them with empty cytoplasmic vacuoles (Figure 1A and B; 63× objective; scale bar: 10 μm). Other cells were eosinophil (1%) and basophil (2%) granulocytes. Oil Red O staining highlighted lipid inclusions in half of the macrophages (Figure 1C and D; 63× objective; scale bar: 10 μm; panel D picture was obtained using differential interference contrast microscopy).

Other biological explorations were performed to exclude metabolic disease, and mediastinal lymphatic malformation was detected by magnetic resonance imaging (MRI), leading to the diagnosis of primary lymphangectasia (neonatal chylous ascites). Symptomatic treatment consisted of repeated punctures until the age of 6 months. A symphysis was then induced by talc injection in the peritoneum to prevent recurrences.

**FIGURE 1:**
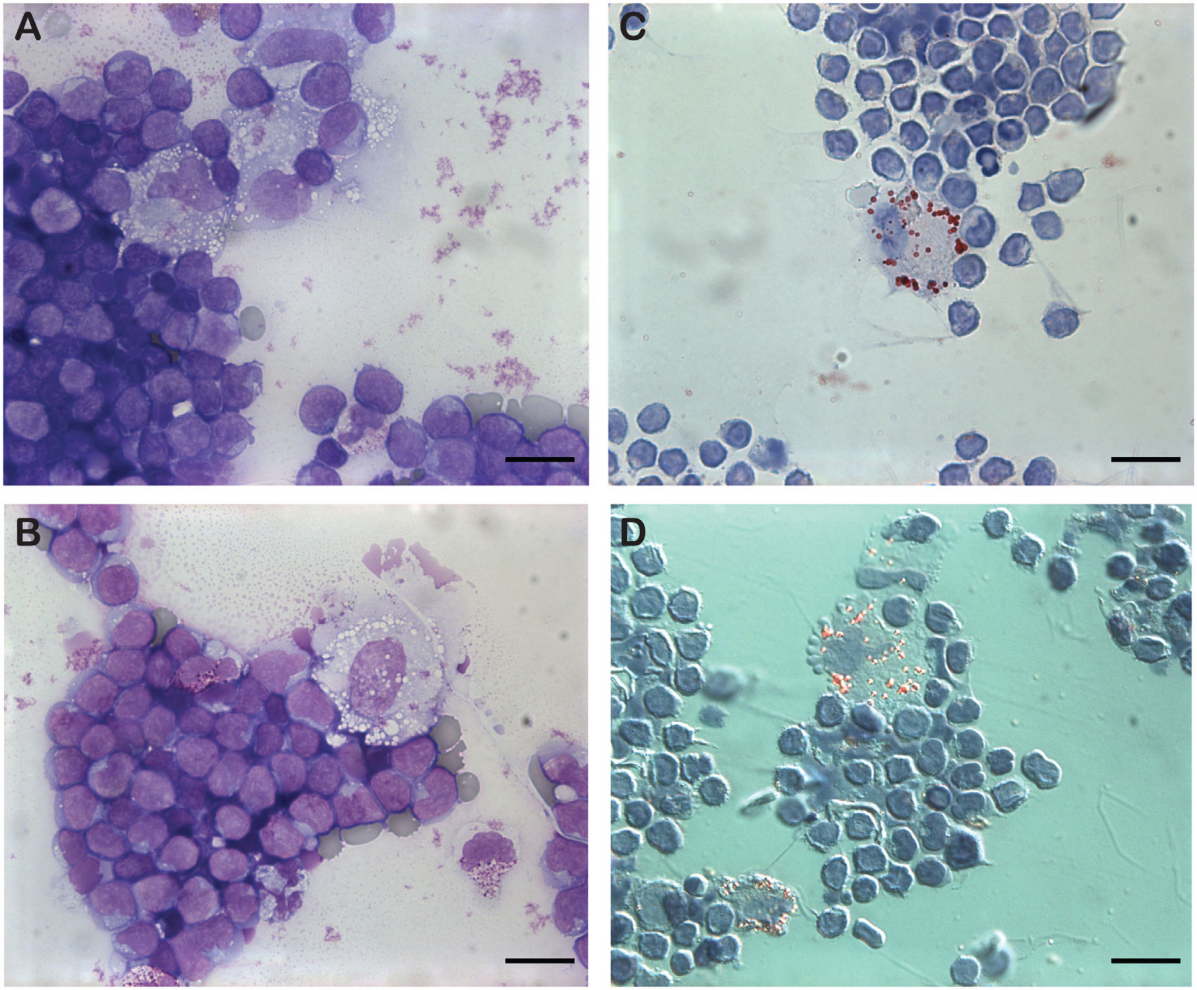
Based on the images provided on the figure, lipid inclusions …: **A**. … are observed in macrophages. **B**. … are observed in lymphocytes. **C**. … are empty after MGG staining. **D**. … appear in red after Oil Red O staining. Response: ACD. Comment: lipids are dissolved with standard stainings (e.g.: MGG or Papanicolaou staining), due to solvents used in these stainings.

## AUTHOR CONTRIBUTIONS

Elise Kaspi, Diane Frankel, and Patrice Roll performed cytological analysis. Elise Kaspi wrote the draft. Diane Frankel and Patrice Roll made corrections and approved the final version.

## CONFLICT OF INTEREST

The authors declare no conflict of interest.

## Data Availability

Data sharing is not applicable to this article as no new data were created or analyzed in this study.

